# Neuroprotective Properties of Linagliptin: Focus on Biochemical Mechanisms in Cerebral Ischemia, Vascular Dysfunction and Certain Neurodegenerative Diseases

**DOI:** 10.3390/ijms20164052

**Published:** 2019-08-20

**Authors:** Michał Wiciński, Karol Górski, Maciej Walczak, Eryk Wódkiewicz, Maciej Słupski, Katarzyna Pawlak-Osińska, Bartosz Malinowski

**Affiliations:** 1 Department of Pharmacology and Therapeutics, Faculty of Medicine, Collegium Medicum in Bydgoszcz, Nicolaus Copernicus University, M. Curie 9, 85-090 Bydgoszcz, Poland; 2 Department of Hepatobiliary and General Surgery, Faculty of Medicine, Collegium Medicum in Bydgoszcz, Nicolaus Copernicus University, M. Curie 9, 85-090 Bydgoszcz, Poland; 3 Department of Pathophysiology of Hearing and Balance System, Faculty of Medicine, Collegium Medicum in Bydgoszcz, Nicolaus Copernicus University, M. Curie 9, 85-090 Bydgoszcz, Poland

**Keywords:** linagliptin, inflammation, pathways, neurodegeneration, pharmacology

## Abstract

Linagliptin is a representative of dipeptidyl peptidase 4 (DPP-4) inhibitors which are registered and used effectively in a treatment of diabetes mellitus type 2. They increase the levels of active forms of endogenous incretins such as GLP-1 and GIP by inhibiting their enzymatic decomposition. Scientific reports suggest beneficial effects of linagliptin administration via immunological and biochemical pathways involved in neuroprotective processes of CNS. Linagliptin’s administration leads to a decrease in the concentration of proinflammatory factors such as: TNF-α, IL-6 and increases the number of anti-inflammatory patrolling monocytes CX3CR1^bright^. Significant reduction in Aβ42 level has been associated with the use of linagliptin implying potential application in Alzheimer’s disease. Linagliptin improved vascular functions by increasing production of nitric oxide (NO) and limiting concentration of apolipoprotein B. Linagliptin-induced decrease in macrophages infiltration may provide improvement in atheromatous plaque stabilization. Premedication with linagliptin increases neuron’s survival after stroke and augments neuronal stem cells proliferation. It seems to be connected with SDF-1α/CXCR4 signaling pathway. Linagliptin prevented abnormal proliferation and migration of rat brain microvascular endothelial cells in a state of hypoperfusion via SIRT1/HIF-1α/VEGF pathway. The article presents a summary of the studies assessing neuroprotective properties of linagliptin with special emphasis on cerebral ischemia, vascular dysfunction and neurodegenerative diseases.

## 1. Introduction

A high percentage of the population struggling with cardiovascular diseases imply a substantial need for new therapeutic methods. Ischemic heart disease and stroke are currently the two most common causes of death worldwide, killing over 15 million people annually [1,2]. The great extent of the problem prompts researchers to seek new properties among medicines already registered in order to optimize treatment. Recent experimental studies indicate that DPP-4 (Dipeptidyl peptidase 4) inhibitor called linagliptin may possess neuroprotective properties. A summary of studies reviewed is presented in the Table 1. DPP-4 inhibitors are registered for treatment of diabetes mellitus type 2. They slow down enzymatic degradation of endogenous incretins such as GLP-1 (Glucagon-like peptide-1) and GIP (Gastric inhibitors polypeptide) and prolong their action. GLP-1 and GIP inhibit the secretion of glucagon from pancreatic alpha cells and reduce gluconeogenesis in the liver. They support the biosynthesis of insulin and its release from pancreatic beta cells. Moreover, incretins reduce the absorption of glucose and delay gastric emptying, which increases the time of satiety and promotes the restriction of food intake [3,4,5]. Above-mentioned antihyperglycemic properties lead to a reduction in fasting blood glucose, postprandial glycemia and HbA1c plasma levels [6,7].

Linagliptin (8-[(3R)-3-aminopiperidin-1-yl]-7-but-2-ynyl-3-methyl-1-[(4-methylquinazolin-2-yl) methyl]purine-2,6-dione) is rapidly absorbed after oral administration and reaches/achieves maximum plasma concentration after approximately 90 min. The absolute bioavailability of 10 mg linagliptin tablet was estimated to be about 30% [8]. Accumulation half-life is only 10 h with 5 mg dosing. Unlike the other DPP-4 inhibitors, 85% of the drug is eliminated unchanged in the feces and only 5% is metabolized by the kidneys and excreted in urine so it can be safely used in patients with kidney diseases [9]. Food intake does not appear to have a relevant influence on linagliptin’s pharmacokinetics [10]. There are no requirements for dose adjustment on the basis of age, sex or weight or due to liver or renal dysfunction [8]. Linagliptin is a weak inhibitor and a weak substrate for CYP3A4 (Cytochrome P450 3A4), but no clinically significant interactions with other drugs have been demonstrated [9].

## 2. Inflammation and Oxidative Stress

Many diseases of the cardiovascular and central nervous system are associated with the presence of inflammation. Acute ischemic event leads to elevation of inflammatory markers concentration such as IL-6 (interleukin 6), CRP (C-reactive protein) and TNF-α (tumor necrosis factor- α) [27]. Monocytes, the precursors of macrophages, are crucial modulators of inflammation in the acute phase of stroke. The activated monocytes have been defined as either classic (M1) or alternative (M2). More precisely, M1 type secretes pro-inflammatory cytokines (TNFα, IL-1β, IL-6, IL-12, IL-23) and exacerbates neuronal injury, whereas the M2 type releases anti-inflammatory agents (TGF-β, IL-4, IL-10, IL-13) and growth factors such as VEGF (vascular endothelial growth factor), BDNF (brain-derived neurotrophic factor) and PDGF (platelet-derived growth factor) which suppresses inflammation, and promotes tissue recovery [28,29,30,31]. Yamadera et al. (2018) in their in vitro study demonstrated a reduction of LPS-induced (lipopolysaccharide) inflammation after linagliptin administration. The production of IL-6 and TNF-α by pro-inflammatory monocytes was significantly reduced in relation to the control sample [32]. Increased levels of IL-6 and TNF-α in both CSF (cerebrospinal fluid) and serum have been associated with deterioration of neurological functions, increased infarct size and poor functional outcome [16,33,34]. Nakamura et al. focused on the assessment of inflammatory parameters of endothelial cells of the umbilical vein exposed to LPS. Linagliptin significantly reduced IL-6 production, p65 expression (member of family NF-κB) and p38 MAPK (mitogen-activated protein kinase) phosphorylation [25]. In 2016, they expanded the spectrum of research with further biochemical parameters such as: ROS/Cu, Zn superoxide dismutase (SOD) index, adenosine 3′,5′-cyclic monophosphate (cAMP) level, protein kinase A (PKA), protein kinase B (PKB) and protein kinase C (PKC) ratio [14]. The ROS/Cu SOD index was determined as an indicator of oxidative stress. Elevated cAMP level induces PKA activation via inhibiting the phosphodiesterase. PKA inhibits MAPK phosphorylation and the subsequent activation of p38. MAPK is involved in maintenaning synaptic plasticity, whereas the elevation of cAMP level concomitantly suppresses NF-κB (nuclear factor kappa B) signaling which is considered to be a prototypical proinflammatory pathway [15,35,36]. Activated PKB and PKC mediate inflammatory responses. Linagliptin has been shown to reduce phosphorylation of PKB in LPS-exposed endothelial cells, however parameters such as: cAMP level, the activity of PKA and surprisingly PKC remained elevated. Dai et al. (2014) demonstrated that two other DPP-4 inhibitors, sitagliptin and vildagliptin limited inflammation response by inhibiting phosphorylation of PKC [37]. Similar effects were expected after the use of linagliptin. Results of Nakamura et al. [15] suggest that PKC may not be involved in the anti-inflammatory effects of linagliptin. It would be interesting to test the other DPP-4 inhibitors in the context of inflammation induced by PKC.

Apart from inhibiting proinflammatory monocytes differentiation, linagliptin was shown to promote the growth of anti-inflammatory cell populations, i.e., non-classic patrolling monocytes. It is a group of phagocytic cells (CX3CR1^high^, CCR2^neg^, Ly6C^low^ in mouse, CD14^low^ CD16^+^ in human) able to migrate along the vascular endothelium. Fadini et al. (2016) demonstrated that 4-day linagliptin supplementation in type 2 diabetic patients leads to a significant increase in plasma patrolling monocytes CX3CR1^bright^ compared to placebo [38]. It has been observed that the patrolling cell may exhibit neuroprotective abilities. In response to bacterial infection patrolling monocytes produced a small quantity of pro-inflammatory cytokines, but significant amounts of anti-inflammatory and healing agents such as IL-1 receptor antagonist, IL-10R, apolipoprotein A (ApoA), apolipoprotein E (ApoE), and CXCL16 (C-X-C motif chemokine ligand 16) [39]. They were proved to be effective in preventing excitotoxity and death of nerve cells as well as in maintaining the integrity of BBB (blood–brain barrier) [40,41].

The endothelial lining can be viewed as the first line of defense between risk factors and vascular disease. This single layer of vascular cells plays a key role in controlling blood hemostasis [42,43]. It is responsible for the synthesis of major vasodilators such as prostaglandins, nitric oxide (NO) and endothelium-derived hyperpolarizing factor (EDHF), as well as endothelin vasoconstrictors (ET) and angiotensin II. After the initiation of inflammatory response, endothelium releases large amounts of ROS. In moderate concentrations they have an important signaling function. It is also well-known that their overproduction is detrimental to the body [44]. Many systemic diseases such as diabetes or arterial hypertension are connected with excessive ROS creation and subsequent vascular injury [45]. Risk factors of atherosclerosis such as hyperlipidemia, diabetes mellitus, hypertension, and cigarette smoking are related to the damaged endothelium. Although, the pathomechanisms are multifactorial, the crucial one is the dysfunction of eNOS/NO pathway that includes the decrease in activity and expression of eNOS (endothelial nitric oxide synthase) and reduced NO sensitivity [46,47].

Nitric oxide is mainly produced from L-arginine by eNOS. NO protects against ischemic stroke by increasing blood flow in the hypoperfusion areas of the brain and in case of the incident, prompts angiogenesis. NO has also been shown to retard the process of SMCs migration from the media to the neointima during atherosclerotic plaque formation [48,49]. Systemic diseases such as diabetes impair NO-dependent muscle relaxation and endothelium dependent hyperpolarization (EDH). They intensify the expression of Nox2, which takes part in the pathogenesis of atherosclerosis and reduces eNOS dimerization [17,50]. Linagliptin seems to promote the normalization of these pathological phenomena. Numerous independent studies proved linagliptin’s influence on NO production and subsequent oxidative stress limitation [17,51,52]. Salheen et al. (2015)dowiedli, że linagliptyna działa protekcyjnie na śródbłonek tętnic krezkowych u szczurów z cukrzycą typu 1 niezależnie od GLP-1/GLP-1R.(2015) demonstrated that linagliptin has a protective effect on the mesenteric artery endothelium of rats with type 1 diabetes mellitus. The effect did not appear to be connected with GLP-1/GLP-1R signaling. Vellecco et al. (2016) potwierdzili, że linagliptyna wpływa na modulacje eNOS niezależnie od GLP-1.(2016) confirmed that linagliptin affects eNOS modulations independently of GLP-1. Badania molekularne komórek śródbłonka dowiodły, że przez blokowanie wiązania eNOS/ caveolin-1 (CAV-1) zwiększa ona dostępność eNOS, co skutkujewzmożonąprodukcją NO [53]. Molecular studies in endothelial cells have shown that linagliptin increases the availability of eNOS by blocking the binding of eNOS and caveolin-1 (CAV-1) leading to enhanced NO production [17].

## 3. Cerebral Blood Flow

Atherosclerosis and its sequelae, such as myocardial infarction and stroke, represent the leading cause of mortality worldwide [54]. One of the earliest detectable changes in the development of atherosclerosis is the activation and dysfunction of endothelial cells in areas prone to arterial damage [55]. In bends and at branch points, blood flow dynamics are disturbed and endothelial cells in this area exhibit a pro-inflammatory and prothrombotic phenotype with reduced barrier function [56]. During the disease the artery wall thickens as a result of the accumulation of cholesterol, macrophages and smooth muscle cells [57]. Progressive atherosclerotic lesions are characterized by a fibrous cap overlapping the lipid-rich necrotic core and the accumulation of leukocytes at the lateral margins, which promotes platelet instability by modulating the EC (endothelial cells) phenotype [53]. The most common clinical complication of atherosclerosis occurs upon plaque lipids and tissue factor, resulting in thrombus formation [58]. Thrombus located in the arteries supplying blood to the brain disturbs its perfusion. Chronic brain hypoperfusion leads to microglia activation poprzez szlak sygnałowyp38MAPK/PKCza pośrednictwemMCP-1 (monocyte chemoattractantprotein 1).via the MCP-1/p38MAPK/ PKC (Monocyte chemoattractant protein 1/p38 mitogen-activated protein kinase/ protein kinase C) signaling pathway [59]. The process causes white matter defects and cognitive decline in mice [60]. The increase in MCP-1 concentration can be observed not only in animals, but also in humans with stroke. Metaanaliza przeprowadzona przezGao et.Meta-analysis conducted by Gao et al. (2014) indicates that the MCP- 1-2518A> G polymorphism and serum MCP-1 concentration may be a potential biomarker for the early detection of cerebral ischemia [61]. Interestingly, u osób chorujących na Alzheimera obserwuje się wzrost liczby klasycznych, prozapalnychmonocytów, jako skutek wysokiego stężenia MCP-1 wsurowicy [35].in patients suffering from Alzheimer’s disease an increase in the number of classical pro-inflammatory monocytes is observed as a result of high serum MCP-1 concentration [62]. W badaniach na ludziach dowiedziono, żelinagliptynaprowadzi do redukcji stężenia MCP-1 w surowicy [25]. In human studies, linagliptin has been shown to reduce serum MCP-1 levels [38]. Limitation of p38MAPK/PKC pathway activation may be a potential mechanism of new therapeutic strategies in cerebral ischemia management. In atherosclerotic mice Salim et al. (2016) have shown that linagliptin decreased MCP-1, VCAM-1 (Vascular cell adhesion protein 1), macrophage marker F4/80 and the expression of the NADPH, p47^phox^ and Nox2 oxidase subunits [22]. Diminished expression of chemokines and adhesion molecules may attenuate macrophage infiltration in the atherosclerotic plaques, which is responsible for plaque destabilization [63]. In studies conducted by Shigiyama et al. (2015) in patients with type 2 diabetes, 16-week linagliptin treatment improved endothelial function. This effect was partially correlated with observed reduction of Apo B [26]. Apolipoprotein B is a low-density lipoprotein involved in the progression of atherosclerosis by reducing nitric oxide-dependent relaxation of artery walls [64]. Meta-analysis published by Dong et al. (2015) implied that increased level of Apo B is associated with an earlier onset of the first ischemic stroke as a consequence of atherosclerosis [65]. Furthermore, linagliptin reduced elevated level of endothelin-1 in DM2 (diabetes mellitus type 2) patients improving the relaxation of blood vessels.

Linagliptin seems to exhibit antagonistic properties towards TLR-2s (Toll-like receptors 2) [23]. TLR-2s serve an essential role in modulating the immune response of the brain [66]. Their expression can be observed on the surface of microglia, astrocytes, neurons and endothelium. These cells secrete pro-inflammatory and pro-apoptotic factors associated with TLR-2 leading to exacerbation of brain damage [67]. Interestingly, it was shown that the area of cerebral infarction in TLR-2 deficient mice was significantly smaller than in the wild type [68]. Lv et al. observed increased expression of TRL2, IL-23, IL-17 following brain ischemia and reperfusion. Microglia in response to ischemia secreted IL-23 and IL-17 leading to neuronal damage. Suppression of the TLR-2-IL23-IL17 axis resulted in the limitation of apoptosis of neurons following ischemia and reperfusion [69]. Linagliptin inhibited the expression of TLR2 resulting in the reduction of cerebrovascular hyperresponsiveness [23].

In the acute phase of stroke necrotic brain tissue in the area supplied by the occluded vessel is surrounded by stressed and vulnerable to death ischemic zone called penumbra. Without therapeutic intervention, many cells within the penumbra will eventually die and become part of the growing infarct. As such, preserving the penumbra to decrease functional defects and promote functional brain recovery is a central goal in stroke research [70,71,72,73]. The results of studies conducted by Zhang et al. (2016) suggest that type 2 diabetes exacerbates vascular and nerve damage and hinders brain repair processes, which probably contributes to impaired performance after stroke [74]. Pre-medication with the use of linagliptin before ischemic event led to an increase of 30% in the survival of neurons in both DM2 and healthy mice. The same antidiabetic drug, glimepiride, exhibited the same effect, but only in healthy mice [18]. Ma et al. (2015) observed that the administration of linagliptin after an episode of transient cerebral ischemia counteracts the cognitive impairment of mice with diabetes. It limited the extravasation of cerebral IgG (Immunoglobulin G) and reduced the excessive activity of microglia as well as inhibited gp91phox of the main subunit of NADPH oxidase responsible for the oxidative stress induction. What is more, there occurred increased concentration of claudin 5 being the essential protein in blood brain barrier functioning [12].

In another study, Darsalia et al. (2014) have observed the beneficial effects of linagliptin on regenerative processes after stroke, but only in mice with DM2. Although this drug did not affect neurogenesis and gliogenesis, it significantly increased the proliferation of stem neural cells that promote the repair of damaged tissues [19]. Darsalia et al. (2016) stated that chronic premedication with linagliptin results in reduction of the ischemic area and increase in the number of preserved neurons after induction of stroke in mice lacking GLP-1Rs (GLP-1 receptors) [75]. Based on the obtained results, the researchers suspected that the action of the DPP-4 inhibitor in the CNS exceeds the mechanism associated with incretin effect [19].

Mi et al. (2018) stated that linagliptin prevents abnormal proliferation and migration of rat brain microvascular endothelial cells (rBMVECs) in hyperglycemic rats under hypoperfusion state. In such conditions, a decrease in the concentration of VEGF, eNOS and HIF-1α (hypoxia-inducible factor-1α) has been observed [13]. Linagliptin treatment ameliorates the adverse effects of hypoxia/hyperglycemia and leads to an increase in abovementioned parameters. HIF-1α regulates the expression of VEGF and eNOS, which serve a key role in the neovascularization process following hypoxia [76,77]. Depletion of SIRT-1 leads to a reduction in the accumulation of HIF-1α, whereas overexpression of SIRT-1 increases the concentration of HIF-1α. SIRT-1 seems to be essential for the stabilization of HIF-1α in a state of hypoxia [78,79,80]. The obtained effects were lessened after SIRT-1 inhibition. Based on the above results, it can be postulated that the protective role of linagliptin on rBMVECs may be mediated by the SIRT1 / HIF-1α / VEGF pathway [13].

Another interesting aspect being contemplated is chemokine 12 (stromal cell-derived factor 1 alpha (SDF-1α)) and its involvement in the maintenance of brain homeostasis. The action of SDF-1α occurs through the activation of CXCR4 and CXCR7 receptors [81,82]. CXCR4 are ubiquitous in the developing nervous system where they participate in neurogenesis and neuronal growth [83]. In AD preclinical and clinical studies decreased SDF-1α levels and CXCR4 expression were observed. Both were associated with adverse cognitive changes [84]. The role of SDF-1α in ischemia of the brain is still under discussion. Some studies show that high levels of SDF-1α correlate with better tissue regeneration [85,86] while others demonstrate that blockade of the SDF-1α/ CXCR4 pathway can cause improvement during recovery [87,88]. Chiazza et al. (2018) showed that treatment with linagliptin increased the concentration of active SDF-1α. There were no changes in the concentration of GIP or GLP-1 in the brain. They observed reduction in the area of stroke and early improvement of the upper limb function previously impaired as a result of ischemia. Furthermore, a lower concentration of basic myelin protein and neurogranin were noted, which is believed to correlate with the area of stroke [89]. The inhibition of the SDF-1α/CXCR4 pathway has eliminated the above-mentioned positive effects. It is known that the concentration of basic myelin protein and neurogranin is regulated by the activity of clapine and intracellular Ca^2+^. It can therefore be assumed that the neuroprotective effects of linagliptin are mediated by the SDF-1α/CXCR4 pathway, which can regulate Ca^2+^ homeostasis and decrease the activity of the calpain protein [24]. A 2-year, randomized, double-blind, non-inferiority trial including 1,552 patients by Gallwitz et al. [90] indicated that patients treated with linagliptin had significantly fewer non-fatal stroke than those treated with glimepiride. On the other hand, results of the studies assessing linagliptin’s efficacy in humans are not consistent. Yang-Rong et al. [91] study on 1203 patients with DM2 results did not find a significant anti-stroke effect with linagliptin treatment at the final follow-up. The actual reason for this discrepancy is unclear but authors postulated that it is due to different study population. There are two linagliptin clinical trials ongoing: CAROLINA^®^ [92] and CARMELINA^®^ [93] which may help to find a conclusion about linagliptin’s efficacy in preventing cardiovascular events in humans.

## 4. Neurodegeneration

Alzheimer’s disease is a progressive neurodegenerative disorder and the most common cause of dementia [94]. On the histopathological level, AD is connected to the deposition of residues consisting of beta amyloid, which are derived from the larger APP (amyloid protein precursors), and NFTs (neurofibrillary tangles) being aggregates of tau protein which binds to microtubules [95]. The process leads to extensive neuronal degeneration and progression of neurological deficits [96,97]. Rising incidence of dementia imposes a search for new therapeutic solutions with a potential to reduce the unfavorable aggregation. Results of experimental in vitro and in vivo studies utilizing linagliptin are promising. Most interestingly, linagliptin’s action may be connected with insulin signaling enhancement as well as be direct and insulin-independent.

The role of insulin in the central nervous system is less known in comparison to peripheral tissues. Recent studies proved the expression of IR (insulin receptors) in neurons and to a lesser extent in glia [98]. Insulin and IGF (insulin-like growth factor) take part in regulation of cellular growth and survival, neuronal stem cell activation, dendritic sprouting, repair, synaptic maintenance as well as in processes involved in learning and memory [99,100,101,102]. Insulin resistance lowers the expression of Aβ-degrading insulin degrading enzyme (IDE) [103]. Rising concentration of Aβ oligomers leads to the removal of IRs from the cell surface via the actions of casein kinase 2 (CK2) and calmodulin II dependent Ca2 + kinase (CaMKII) turning the sequence of events into a vicious circle [104]. Limitation in brain insulin signaling increases GSK-3β (Glycogen synthase kinase 3) activity. The process leads to abnormal tau protein phosphorylation [105]. Enhancement of insulin signaling may be a substantial molecular target in linagliptin’s action. Kornelius et al. (2015)w badaniach na komórkach neuronalnych wykazali, że linagliptyna działała ochronnie przeciwko cytotoksyczności.(2015) have shown that linagliptin exhibits some protective properties in this aspect. By restoring proper insulin signaling it was able to prevent GSK3β activation and by that potentially affect tauopathy [102]. In mice model of AD, Kosaraju et al. (2017)(2017) achieved statistically significant reduction in the level of Aβ42 without affecting the levels of Aβ40. Aβ42 compared to Aβ40 is preferentially deposited in the extracellular space where creates plaques of the protein [21]. Some control of the next characteristic neuropathological hallmark of AD-tauopathy has been also obtained. The number of NFTs is tightly linked to the degree of dementia, suggesting that the formation of neurofibrillary tangles more directly correlates with neuronal dysfunction [106]. It has been reflected in the results. Reduction in plaque load and NFTs deposition have improved cognitive functions of linagliptin-treated mice [21].

The disproportion between pro-inflammatory and anti-inflammatory factors correlates with an increased ryzykiemzgonu [10].d risk of death and has been linked to development of neurodegenerative diseases such as Alzheimer’s disease [5,106,107,108,109]. Systemic inflammation may disturb the integrity of blood–brain barrier leading to migration of proinflammatory agents to the CNS [110]. Consequent chronic low-grade inflammation has been proven to promote the development of neurodegenerative diseases [111]. Research conducted by Elbaz et al. (2018) dowodzą,żeleczenie linagliptyną poprawiafunkcje poznawcze i motoryczne u myszy z demienilizacją indukowaną cuprizonem.argue that treatment with linagliptin improves cognitive and motor functions in cuprizone-induced demyelization. The observed anti-inflammatory effect of linagliptin resulted from a decrease in NF-κB concentration. Epidemiological studies show that the brain of patients with AD has a higher concentration of NF-kB. Aforementioned factor is able to NF-κB może promować apoptoze komórek oraz brać udział w amyloidogenezie poprzez indukcję ekspresji APPpromote cell apoptosis and participate in amyloidogenesis by inducing expression of APP (Amyloid Precursor Protein)i BACE1(Amyloid Precursor Protein) and BACE1 (Beta-site amyloid precursor protein cleaving enzyme 1). (Beta-site amyloid precursor protein cleaving enzyme 1). What is more, the reduction of NF-κB obtained by chronic administration of non-steroidal anti-inflammatory drugs inhibits the progression of AD and delays its consequences [98]. JAK/STAT/NF-κB (The Janus Kinase/Signal Transducers and Activators of Transcription/nuclear factor kappa B) pathway serve an important role in the development of both congenital and acquired immunity [20]. Głęboko zaangażowany w ten proces jest także szlak NF-κB (nuclear factor kappa B). Due to their interconnection, they were shown to play an important role in the pathogenesis of inflammatory neurodegenerative diseases such as multiple sclerosis - chronic demyelinating disease of the central nervous system [112,113]. Various molecular factors are prone to promote inflammatory response by JAK2 phosphorylation and transcription of STAT3. Aktywacjatego szlaku w mikrogleju prowadzi do indukcji transkrypcji prozapalnych cytokin oraz białek regulatorowych takich jak IL-1/6, TNF-alpha, MIFActivation of this pathway in microglia leads to the induction of transcription of proinflammatory cytokines and regulatory proteins such as IL-1, IL-6, TNF-α, IFN-γ and MIF (macrophage migration inhibitory factor), IFN-gamma [38].(macrophage migration inhibitors factor) [114]. Opposite effects in relation to the JAK2/STAT3/NF-κB pathway are correlated to AMPK/SIRT1 signaling pathway activation. AMP-activated protein kinase (AMPK) serves a major role in maintaining cell energy homeostasis [115]. It regulates inter alia: glucose and fatty acids uptake, synthesis of cholesterol, triglycerides, glycogen and proteins, glycolysis and mitochondrial biogenesis. Its expression may favorably affect the insulin sensitivity of tissues, particularly in patients with type 2 diabetes [116]. Many authors suggest that the AMPK/SIRT pathway plays an essential role in preventing and limiting the progression of demyelinating and inflammatory diseases of the CNS [117,118]. SIRT1 (NAD-dependent deacetylase sirtuin-1) belongs to the sirtuin family involved in the regulation of gene expression and metabolic changes [119]. It is supposed that linagliptin mitigates the mitochondrial dysfunction and reduces excessive ROS production caused by Aβ via the AMPK/SIRT1 pathway activation [11]. Increased SIRT1 activation was observed as a result of linagliptin administration both in animals and patients suffering from DM2. Based on the above observations, it can be assumed that described neuroprotective effect of linagliptin may result from the inhibition of the JAK2/STAT3/NF-κB pathway in favor of the stimulation of the AMPK/SIRT1 pathway [20].

Linagliptin may support the clearing of pathological aggregations in AD. Michaud et al. (2013) have noticed that CX3CR1 patrolling monocytes are attracted to vessels within which amyloid beta deposits occur.depozyt AThey managed to observe, for the first time in vivo, that abovementioned subtype of monocytes possesses the ability to phagocytize endogenous Aβ residues. CX3CR1 patrolling monocytes are able to return to systemic circulation and by that reduce Aβ concentration in cerebral vascular space [102]. Linagliptin proved to have some potential to increase CX3CR1 monocyte population which may represent another mechanism of its action [38]. Interesującą kontynuacją powyższych odkryć byłoby przeprowadzeniebadań klinicznych oceniających wpływ linagliptyny na populację CX3CR1 uludziz AD. An interesting continuation of the above findings would be to conduct clinical trials assessing the effect of linagliptin on the CX3CR1 population in people with AD.

**Figure 1:**
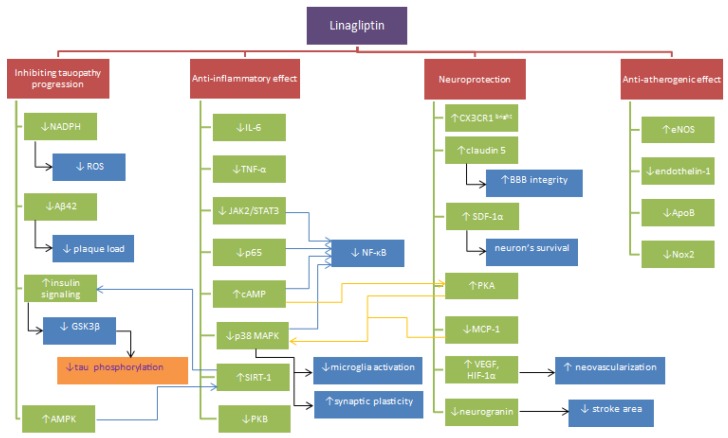
Proposed mechanisms of linagliptin activity. Note: ↓ = reduction, ↑ = increase, SDF1 = stromal cell-derived factor 1, CX3CR1^brigh^- monocytes type, *MCP-1* = Monocyte chemoattractant protein-1, *JAK2* = Janus kinase 2, AMPK = 5′AMP-activated protein kinase, STAT3 = *Signal transducer and activator of transcription*, NF-κB p65 = nuclear factor kappa-light-chain-enhancer of activated B cells, SIRT1 = NAD-dependent deacetylase sirtuin-1, Aβ = amyloid beta, GSK3β = Glycogen synthase kinase 3 beta, ROS = reactive oxygen species, tau = tau protein, NO = nitric oxide, NADPH = Nicotinamide adenine dinucleotide phosphate, Nox2 = NADPH oxidase 2, IL-6 = *Interleukin 6, TNF-α = tumor necrosis factor α*, p38 MAPK = P38 mitogen-activated protein kinases, PKA = *protein kinase* A, PKB = *protein kinase* B, cAMP = 3′,5′-cyclic adenosine monophosphate, Apo B = *Apolipoprotein B*, VEGF = vascular endothelial growth factor, eNOS = endothelial nitric oxide synthase, HIF-1α = *hypoxia-inducible factor 1*, p65 = subunit of NF-κB.

Beneficial effects of linagliptin’s applications may be due to hypoglycemic properties itselves. Chronic hyperglycemia and insulin resistance promote the formation of CNS injuries as a result of impaired mitochondrial function [120], induction of inflammation [121], free radicals’ overproduction [120], promotion of apoptosis [122], dysfunctions of the blood-brain barrier [88] and tau metabolic pathway [123]. In their review, Bruno et al. (2004) described implications of high glucose concentration stating that it exacerbates ischemia/reperfusion-related brain injury in animals [124] and correlates with increased mortality after acute ischemic stroke [125]. A high level of glucose increases the sensitivity of endothelial cells of cerebral microcirculation to Aβ toxicity promoting brain damage in the course of AD [126]. It is supposed that these processes are consequent to losing the integrity of the blood-brain barrier by degradation of tight junction protein [127]. As a result of exposure to hyperglycemia endothelial cells show overproduction of ROS and associated advanced glycation end products (AGE). The increase in the concentration of α-dicarbonyl glucose metabolites (AGE precursors) and AGE-receptors disturbs monocyte polarization in favor of the inflammatory phenotype. The process contributes to damage enlargement [121]. A lot of evidence indicates that AGEs serve very important role in the development of vascular complications in the course of DM, which may lead to the emergence of AD [128]. DM2 promotes deposition of the cerebral amyloidβ and the pancreatic human islet amyloid polypeptide (hIAPP). The aggregation causes increased phosphorylation of tau protein and reduction of β-cell size in pancreatic islets responsible for insulin secretion [128]. Limitation of such consequences provided by linagliptin may prove to be useful. Figure 1. presents the proposed mechanisms of linagliptin’s action summarized in the paper.

## 5. Conclusions

Reviewed studies allow for the consideration that the effects of linagliptin are beyond glycemic control. Presented findings introduce linagliptin as a promising drug in the treatment of DM2 patients with vascular and neurological disorders. If DPP-4 inhibitors are demonstrated to have clinically meaningful anti-sclerotic activity in humans, one potential application may be to reduce the burden of certain neurodegenerative disorders. The moderation of ROS production and aggregation of *β*-amyloid may prove to be helpful in AD, stroke and changes related to chronic hyperglycemia of the central nervous system. Most of the studies were conducted on cell lines and animal models, so validation of presented mechanisms requires a multidimensional verification regarding both the dosage of the drug and its effect in humans. Due to many methodological limitations of the above-mentioned studies, it is essential to perform more randomized and double-blind placebo-controlled clinical trials to prove the clinical utility of linagliptin in the discussed conditions.

## Figures and Tables

**Table 1:** Summary of reviewed results. Note: ↓ = reduction, ↑ = increase, Aβ = amyloid beta, GSK3β = Glycogen synthase kinase 3 beta, ROS = reactive oxygen species, p- = *phosphorylation*, tau = tau protein, rBMVECs = rat Brain Microvascular Endothelial Cells, BCCAO = Bilateral Common Carotid Artery Occlusion, *COS= cerebral oxygen species*, VEGF = *vascular endothelial growth factor*, eNOS = endothelial nitric oxide synthase, HIF-1α = *hypoxia-inducible factor 1*, SIRT1 = NAD-dependent deacetylase sirtuin-1, HUVECs = Human Umbilical Vein Endothelial Cells, LPS = Lipopolysaccharides, IL-6 = *Interleukin 6*, p38 MAPK = P38 mitogen-activated protein kinases, NF-κB p65 = nuclear factor kappa-light-chain-enhancer of activated B cells, PKA = *protein kinase A, PKC = protein kinase C*, cAMP = 3′,5′-cyclic adenosine monophosphate, *PKB = protein kinase B, TNF-α = tumor necrosis factor α*, NO = nitric oxide, EDR = endothelium-dependent relaxation, NADPH = Nicotinamide adenine dinucleotide phosphate, Nox2 = *NADPH oxidase 2*, MCAO = middle cerebral artery occlusion, NSCs = neuronal stem cells, *JAK2 = Janus kinase 2*, AMPK = 5′AMP-activated protein kinase, STAT3 = *Signal transducer and activator of transcription*, VCAM-1 = *vascular cell adhesion molecule 1, MCP-1 = Monocyte chemoattractant protein-1, ET1 = Endothelin 1*, TLR2 = *Toll-like receptor 2, SDF1 = stromal cell-derived factor 1*, CX3CR1^brigh^- monocytes type, *CCL22-* C-C motif chemokine 22, IL-12 = *Interleukin 12*, Apo B = *Apolipoprotein B*.

Authors	Subject of Study	Dose of Linagliptin	Results
Kornelius et al. (2015) [11]	SK-N-MC human neuronal cells	10–100 μM of linagliptin for 24 h	↓ Aβ-induced cytotoxicity, ↓ GSK3β, ↓ ROS, ↓ hyper p-tau
Ma et al. (2015) [12]	rBMVECs	0.083 g/kg diet for 8 weeks ater BCCAO	↓ cognitive impairment, ↓ stroke volume, ↓ COS
Mi et al. (2018) [13]	rBMVECs	40 nM	↑ VEGF, ↑ eNOS, ↑ HIF-1α, ↑ SIRT1,
Nakamura et al. (2016) [14]	HUVECs	1, 5, 10, 50, and 100 nM 1 h prior to incubation with LPS	↓ IL-6, ↓ p-p38 MAPK ↓ p65
Nakamura et al. (2016) [15]	HUVECs	1 h 50 nM after 1 h 1 μg/mL LPS together with 50 nM linagliptin,	↑ PKA, ↑ PKC, ↑ cAMP, ↓ PKB ↓ ROS
Yamadera et al. (2018) [16]	U937 cells	1, 5, 10, 50, or 100 nM	↓ IL-6, ↓ TNF-α
Salheen et al. (2015) [17]	STZ-induced diabetic rats	2 mg/kg/ day for 4 weeks	↑ NO, ↑ EDR, ↓ NADPH, ↑ Nox2
Darsalia et al. (2013) [18]	C57BL/6 mice	10 mg/kg/day for 4 weeks before and 3 weeks after MCAO	↑ survival of neurons
Darsalia et al. (2014) [19]	C57BL/6 mice	10 mg/kg/day for 4 weeks before and 3 weeks after MCAO	↑ NSCs proliferation
Elbaz et al. (2018) [20]	C57BL/6 mice	10 mg/kg/day for 3 weeks after 2 (from 3) weeks cuprizone administration	↓ p-JAK2, ↑ p-AMPK, ↓ p-STAT3, ↓ NF-κB p65, ↑ SIRT1.
Kosaraju et al. (2017) [21]	3xTg-AD mouse	5, 10, and 20 mg/kg/day for 8 weeks.	↑ Cognitive Performance, ↓ Aβ42, ↓ hyper p-tau
Salim et al. (2016) [22]	ApoE(−/−) mice	10 mg/kg/day for 20 weeks	↓ VCAM-1 ↓ MCP-1 ↓ NADPH
Hardigan et al. (2016) [23]	Male type-2 diabetic GK rats	83 mg/kg for one week, next 166mg/kg for three weeks	↓ ET-1, ↓ TLR2
Chiazza al. (2018) [24]	C57BL/6 mice	varied at every stage of the experiment	↑ post stroke rehabilitation ↑ SDF-1α ↓ stroke volume
Fadini et al. (2016) [25]	Diabetes type 2 patients	5 mg per day for 4 days	↑ SDF-1α, ↑ CX3CR1^bright^, ↓ MCP-1, ↓ CCL22, ↓ IL-12
Shigiyama et al. (2015) [26]	Diabetes type 2 patients	750 mg/day metformin + 5 mg/day linagliptin for 16 weeks	↓ Apo B
